# Integrating Next-Generation Sequencing in the Clinical Pharmacogenomics Workflow

**DOI:** 10.3389/fphar.2019.00384

**Published:** 2019-04-05

**Authors:** Efstathia Giannopoulou, Theodora Katsila, Christina Mitropoulou, Evangelia-Eirini Tsermpini, George P. Patrinos

**Affiliations:** ^1^Department of Pharmacy, School of Health Sciences, University of Patras, Patras, Greece; ^2^The Golden Helix Foundation, London, United Kingdom; ^3^Department of Pathology, College of Medicine and Health Sciences, United Arab Emirates University, Al-Ain, United Arab Emirates; ^4^Zayed Center of Health Sciences, United Arab Emirates University, Al-Ain, United Arab Emirates

**Keywords:** clinical pharmacogenomics, workflow, implementation, next-generation sequencing, clinical decision support tools

## Abstract

Pharmacogenomics has been recognized as a fundamental tool in the era of personalized medicine with up to 266 drug labels, approved by major regulatory bodies, currently containing pharmacogenomics information. Next-generation sequencing analysis assumes a critical role in personalized medicine, providing a comprehensive profile of an individual’s variome, particularly that of clinical relevance, comprising of pathogenic variants and pharmacogenomic biomarkers. Here, we propose a strategy to integrate next-generation sequencing into the current clinical pharmacogenomics workflow from deep resequencing to pharmacogenomics consultation, according to the existing guidelines and recommendations.

## Introduction

Since the 1950s, many pioneers in the biomedicine field have reported individual variability in disease management and envisioned personalized medicine in health care (Evans and Relling, 1999). Notwithstanding, the realistic application of genomic findings and technologies in the clinic goes beyond the discovery of gene variants and their validation in clinical trials. Lam (2013) has suggested a series of stages regarding the development and implementation pathways for pharmacogenomic tests, namely: (i) discovery of pharmacogenomic biomarkers and validation in well-controlled studies with independent populations; (ii) replication of drug-gene(s) association and demonstration of utility in at-risk patients; (iii) development and regulatory approval of companion diagnostic test; (iv), assessing the clinical impact and cost-effectiveness of the pharmacogenomic biomarkers; (v), involvement of all stakeholders in clinical implementation (Lam, 2013).

Noteworthy, the scientific challenges and implementation barriers existing within the abovementioned stages are still rather unmet. Pharmacogenomic testing occurs by genotyping or sequencing and is mostly outsourced from hospitals to private companies, being a time-consuming and costly process (Harper and Topol, 2012). Unfortunately, there is still a profound lack of understanding within the medical community regarding genomics and the impact of genomic variants in rationalizing drug prescription (Stanek et al., 2012; Mitropoulou et al., 2014). On the other hand, the pharmacy benefit managers (involved with authorizing of fulfilling most prescriptions in the United States) have been particularly interested in the use of pharmacogenomic testing to save employers (their customers) the cost of a drug through genotyping, making the pharmacy benefit managers in question more competitive (Topol, 2010).

In 2013, the United States Food and Drug Administration (FDA) announced a guidance for industry entitled “Clinical Pharmacogenomics: Premarket Evaluation in Early-Phase Clinical Studies and Recommendations for Labeling^1^” in an effort to address the challenges that need to be met. The FDA has also established the Genomics and Targeted Therapy Group^2^ toward the advancement of the application of genomic technologies in the discovery, development, regulation, and use of medications. In the same context, the United States National Cancer Institute has announced a rather similar research and development workflow toward treatment strategies in cancer, including: (i) the support of the routine collection of germline and tumor biospecimens from clinical trials or population-based studies, (ii) the support in efficacy/toxicity biomarker development, (iii) the incorporation of pharmacogenomic markers into clinical trials, and (iv) the consideration of ethical, legal, social, biospecimen, and data-sharing implications of pharmacogenomics research (Freedman et al., 2010). Today, FDA has approved 266 drugs that include genetic information in their labels (Drozda et al., 2018) and the same is true for the European Medicines Agency (Ehmann et al., 2015). The distribution of these drugs between various target diseases indicates that oncology, cardiology, psychiatry, and neurology are among the most common ones in which pharmacogenomics are readily applicable for routine clinical care (Potamias et al., 2014).

## Next-Generation Sequencing Genotyping in Pharmacogenomics

Considering the plummeting cost of genotyping, particularly in a high-throughput format, such as panel-based genotyping and/or next-generation sequencing as well as data accuracy improvements, one would envisage that comprehensive pharmacogenomic testing using these approaches could be readily applicable in a clinical setting (Kitzmiller et al., 2011). Indeed, major academic institutions, government-sponsored as well as private organizations and research consortia are engaged into collaborative programs that focus on next generation sequencing of the cancer genome, aiming to describe the architecture of cancer-specific somatic alterations and as such, aid clinicians toward disease management (Simon and Roychowdhury, 2013), while others, such as the SEAPharm Consortium^3^ are currently exploring the use of targeted pharmacogene resequencing in 100 pharmacogenes to explore the pharmacogenomic variants allelic architecture and the most prevalent pharmacogenomic biomarkers in Southeast Asian populations.

Recently, by investigating the exome sequences or over 60000 individuals, Ingelman-Sundberg et al. (2018) demonstrated that each individual harbors, on average, approximately 41 putatively functional pharmacogenomic variants from which 10.8% are rare and found to be highly gene- and drug-specific, accounting for a substantial part of the unexplained inter-individual differences in drug metabolism phenotypes.

Still, and contrary to identifying the genetic basis of disorders characterized by a high degree of phenotypic and clinical variability and/or genetic heterogeneity (Ku et al., 2016), in case of pharmacogenomic testing, where the role of several pharmacogenes is well established, targeted gene resequencing seems to be perhaps more relevant compared to whole exome sequencing, as it also captures rare pharmacovariants that are present in other genomic positions than the gene exons, such as promoters, intronic and untranslated sequences, which have been shown to lead to drastic reduction of drug metabolizing enzyme activity. This is further highlighted in a recent study comparing the results obtained by whole genome sequencing, whole exome sequencing, and microarray-based genotyping, indicating that the performance of genotyping arrays is similar to that of whole genome sequencing, whereas whole exome sequencing is not suitable for pharmacogenomics predictions (Reisberg et al., 2019). In any case, novel and rare pharmacovariants that can only be identified by next-generation sequencing approaches are of utmost importance in personalized drug therapy to provide information of use to avoid adverse drug reactions and lack of response (Lauschke and Ingelman-Sundberg, 2018).

Tumor samples are known to contain both acquired and inherited alterations, along with somatic DNA. Thus, cancer sequencing efforts also capture germline information. This germline information plays a crucial role in optimizing the dose and selection of therapy. A unique benefit to next generation sequencing is the ability to discover rare variants (in cancer patients, germline DNA is also analyzed as a means to identify variants in the tumor) in the genome and then, delineate their impact on drug response (Gillis et al., 2014). This has been previously demonstrated by Mizzi et al. (2014), indicating that novel and rare variants can exert a deleterious effect in drug metabolizing enzymes, such as CYP2D6, TPMT, CYP2C19, involved in anti-cancer, psychiatric and cardiology drug treatment, among others, by introducing premature stop-codons or out-of-frame frameshifts very close to the N-terminus of the enzyme. These authors also demonstrated that whole genome sequencing could identify novel *CYP2C9* variants relevant to anticoagulation treatment, which could not have been identified using microarray-based genotyping approaches, which could potentially guide toward alternative anticoagulation treatment modalities in two patients suffering from atrial fibrillation (Mizzi et al., 2014). Furthermore, rather than Sanger sequencing, next generation sequencing technology yields more accurate quantitative results, when somatic variation is considered and can be achieved at a higher throughput scale (Simon and Roychowdhury, 2013). Indeed, findings in genes involved in the metabolism of anti-cancer drugs further demonstrate the potential applicability of whole genome sequencing for pharmacogenomic testing in a clinical setting in the not too distant future (McCarty et al., 2011; Mizzi et al., 2014; Karageorgos et al., 2015).

## Information Technologies and Data Interpretation

Currently, difficulties in pharmacogenomics data interpretation are claimed responsible for the slow clinical uptake of pharmacogenomics. Two main aspects of data interpretation have been identified to affect pharmacogenomics translation into clinical practice: (i) the interpretation of reported genetic results by clinicians and (ii) the interpretation of published research results. It has become evident that the vast majority of health professionals even though acknowledges that genetic variations may influence drug response, only a limited number of those feel adequately informed about pharmacogenomic testing and data interpretation (Stanek et al., 2012; Mitropoulou et al., 2014). So far, standardization in conducting pharmacogenomics studies is lacking, mainly due to inconsistencies in results reporting (O’Donnell and Ratain, 2012). These inconsistencies make data interpretation challenging or even chaotic to researchers, professional organizations, consortia and clinicians alike and international efforts are currently ongoing to standardize pharmacogenomics testing reporting (Kalman et al., 2016).

With the advent of next generation sequencing, collaboration toward data accumulation would help maximize its clinical benefit, as large sample sizes would provide the means to retrospectively analyze large patient cohorts for (i) discovery of common and rare variants, (ii) validation, and (iii) pharmacogenomics outcomes toward decision-making. Today, the Electronic Medical Records and Genomics (eMERGE) Network, attempts to maximize the benefit from next generation sequencing analyses, focusing on the combination of DNA biorepositories with electronic medical records to facilitate large-scale, high-throughput genetic research and return genetic testing results to patients in a clinical setting (McCarty et al., 2011). Such efforts would be beneficial to be exploited, including somatic and germline variation discovery and implementation as well as clinical and uptake outcomes.

To this end, in the big data era, biomedicine scientists need to critically appraise data, collaborate in an efficient and effective way and make decisions. For this, large-scale volumes of complex multi-faceted data need to be meaningfully assembled, mined, analyzed and provided in a user-friendly manner. An innovative web-based collaboration support platform that adopts a hybrid approach on the basis of the synergy between machine and human intelligence was previously reported, aiming to facilitate the underlying sense-making and decision making processes (Tsiliki et al., 2014). Clinical decision support (CDS) tools have been also proved valuable in the context of clinical pharmacogenomics, as they provide guidance on clinical decisions, through electronic medical records (Bell et al., 2014). Again, these tools demand clear and precise algorithms based on scientifically robust findings, ideally synergizing among different variant prediction tools to take novel and rare pharmacogenomic variants into consideration to determine their pathogenicity.

## Validation and Accreditation of Services

The application of pharmacogenomics in personalized medicine is very challenging and influence medicine and biomedical research in many areas, namely clinical medicine, drug development, drug regulation, pharmacology, and toxicology (Tremblay and Hamet, 2013; Drozda et al., 2018). However, many issues have to be addressed including genomic data quality and assays’ accreditation.

According to the European Medicines Agency (EMA) guidelines, there is a regulatory framework defined by Good Clinical Practice (DCP) compliance (European Medicines Agency, 2001/2005), Good Laboratory Practice (GLP) compliance (European Medicines Agency, 2015), Good Manufacturing Practice (GMP), and Good Distribution Practice (GDP) (European Medicines Agency, 2001), while recently a guideline for Good Pharmacogenomics Practice has been produced (European Medicines Agency, 2018). In particular, this guideline stresses the importance of all steps included in any next-generation sequencing protocol from DNA extraction, DNA processing, preparation of libraries, generation of sequence reads and base calling, sequence mapping, variant annotation and filtering, variant classification, and interpretation. According to this guideline, a crucial parameter for next-generation sequencing analysis is the minimum sequencing coverage, which in case of germline pharmacovariants should be at least 30×, while in case of rare variants, a higher coverage is needed in order to ensure that also the rarer variants are detected by the sequencing. Also, in case or highly homologs genes and pseudogenes, that can contribute to miscalled variants due to sequencing artifacts, it is recommended to include methods that use substantially longer read lengths, i.e., fragments longer that 1000 base pairs.

This guideline portfolio has been developed to ensure the quality of medical products and services. The transfer of this policy to pharmacogenomics assays is critical, since numerous studies have pointed sources of inter- and intra-laboratory error and variability in experimental results (Ji and Davis, 2006). The quality issues of pharmacogenomics rely on the genomic complexity of the region of interest that can impact accuracy and precision of an assay. Consequently, it is important to understand and give due consideration to assay design (Pant et al., 2014), especially when it comes to next-generation sequencing.

Additionally, the validation of the discovery findings coming from pharmacogenomics studies in large randomized clinical trials is often difficult, due to high costs and ethical considerations (Wheeler et al., 2013). In the case of prospective clinical trials, specific drug-dosing schedules are used, providing consistent and well-maintained drug data for pharmacogenomics studies. To increase the sample size for a particular phenotype, it may be useful to combine data from the treatment arms of a clinical trial and then, control for potential confounding, owing to treatment differences during the statistical analyses. In this context, cancer pharmacogenomics studies have shown promising results, although replication may still be an issue (Hyman et al., 2015). Currently, there are not enough well phenotypic patient data sets for most cancer drugs under investigation to make replication studies feasible, especially when effect sizes are small (Spencer et al., 2009; Daly, 2010). Despite the limitations and difficulties with samples’ size, cancer pharmacogenomics studies have demonstrated the potential to make therapy safer and more effective for patients (Spencer et al., 2009; Daly, 2010; Wheeler et al., 2013).

## Consultation

There is uncertainty about the ways that the results of pharmacogenomics can be translated into clinical care decisions by the government agencies. This reflects the complex genetic interactions, the paucity of evidence (in some cases) as well as the legal constraints by the regulatory bodies. As a consequence, health professionals are in a vulnerable position (Maliepaard et al., 2013; Trent et al., 2013). This status is imprinted by the United States FDA policy that orders every pharmacogenomics product to provide any relative information available, but without any use recommendation (Maliepaard et al., 2013; Trent et al., 2013). Uncertainty and lack of information ask for additional pressure on professional societies to develop the appropriate clinical practice guidelines to ensure that patient care is not compromised or unnecessary genetic testing is avoided (Maliepaard et al., 2013; Trent et al., 2013). No doubt, multiple sources of information on pharmacogenomics tests can create confusion in clinical decision-making. To overcome this, PharmGKB^4^ was established to consolidate datasets into one curated database, where users can query for drug, gene, disease or metabolic pathway to obtain information such as drug properties, pathway diagrams as well as related publications in a centralized manner. Also, the Clinical Pharmacogenetics Implementation Consortium (CPIC^5^) and the Dutch Pharmacogenetics Working Group (Dutch Pharmacogenetics Working Group, 2005) have issued guidelines per gene-drug combination assisting healthcare professional to interpret pharmacogenomic testing results and reciprocally adjust the dose or select an alternative drug.

## Concluding Remarks

In the era of big data and -omics technologies, the translation of pharmacogenomics in the clinic has yet to be met. This does not only refer to next-generation sequencing-based genotyping but also the more easily applicable low-to-medium throughput (single variant to panel-based) genotyping. Nevertheless, next-generation sequencing will soon be part of the clinical reality and as such, one of the first areas that will be readily applicable is the rationalization of drug use.

**FIGURE 1:**
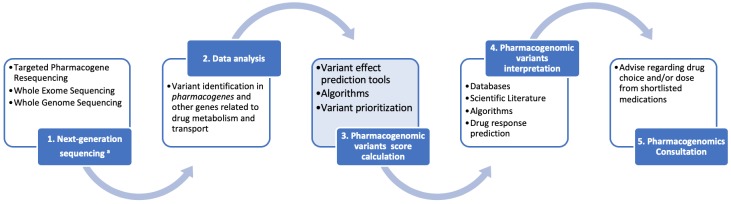
A schematic representation for the clinical pharmacogenomics workflow described herein. We feel that the advent of next generation sequencing (NGS) will accelerate the clinical applications of pharmacogenomics through a series of reliable, cost-effective opportunities. Data collection and interpretation will benefit from the interplay of consortia and information technologies. Regulatory bodies will lead the way toward assay validation and accreditation, considering the difficulties of pharmacogenomics studies replication. Consultation, as the final step of our workflow, facilitates the bench-to-bed transition.

Depending on the available resources and infrastructure, application of next-generation sequencing in pharmacogenomics will vary from targeted pharmacogene resequencing in low resource settings, be it either in a panel-based format per drug categories (e.g., cardiovascular diseases, oncology, psychiatric diseases, etc.) or in a more comprehensive pre-emptive pharmacogenomics format including as many pharmacogenes as possible. In those settings, where whole exome, or – ideally – whole genome, sequencing is available, then pharmacogenomic variant identification will be performed simultaneously with the disease genetic diagnosis, focusing only on those variants in the pharmacogenes. As such, the following workflow is recommended for clinical pharmacogenomics (outlined in Figure 1):

(1) Next generation sequencing (targeted pharmacogene resequencing, whole exome and/or whole genome sequencing) will be performed in duly accredited laboratories, following the established guidelines for good pharmacogenomics and other practices,
(2) Data analysis will follow for the identification of the common, rare and even novel, genomic variants in the pharmacogenes and other genes involved and/or related to drug metabolism and transport, using dedicated data analysis software packages,
(3) An integral part of the pharmacogenomic variant annotation will be the calculation and assignment, to each pharmacovariants, of a specific score. This score will be calculated based on certain criteria, such as: (i) the variant itself [known (well established function) or novel (not functionally validated)], (ii) in case of novel variants or variants of unknown significance, the nature of the variant itself (nonsense, frameshift, non-synonymous, other, etc.), the effect of which will be determined *in silico* by a battery of variant prediction tools, (iii) the variant’s frequency in the population (common or rare), (iv) the existing evidence of the pharmacogene role, in which the variant is identified, in drug metabolism and transport, deducted from the various databases [e.g., Level A–D (for CPIC) or Level 1–4 (for PharmGKB), etc]. Subsequently, variant prioritization will be performed, based on these scores,
(4) After pharmacogenomic variants are prioritized, their interpretation will follow, based on the scientific literature, databases, algorithms, from which the corresponding drug response predictions will be derived, also in conjunction with recommendation resources, such as the CPIC, PharmGKB, or the Dutch Pharmacogenomics Working Group, and lastly,
(5) Pharmacogenomics consultation, performed by a qualified pharmacogenomics expert or clinical geneticist, that will include the provision of advice regarding the drug choice from a shortlist of suggested medications to avoid adverse drug reactions and/or to ensure the optimal drug treatment.

Such a pharmacogenomics scoring system is currently being developed (Patrinos GP, unpublished) to facilitate integration of next-generation sequencing for pharmacogenomics into the routine clinical care. In addition, there are further opportunities for omics-related disciplines, beyond genomics, to be employed for personalized drug response predictions, namely pharmacoepigenomics (Lauschke et al., 2018), pharmacometagenomics (Balasopoulou et al., 2016) and/or pharmacometabolomics (Balasopoulou et al., 2016; Balashova et al., 2018).

We feel that proper implementation of the proposed workflow for next-generation sequencing-based pharmacogenomic testing can occur only via the synergy of all stakeholders and their will to implement the current technological advances, in this case, next generation sequencing and information technologies. In cancer, particularly, such a synergy would be greatly beneficial toward the enigmatic complexity of the disease and great individual variability.

## Author Contributions

All authors have compiled and approved the manuscript.

## Conflict of Interest Statement

The authors declare that the research was conducted in the absence of any commercial or financial relationships that could be construed as a potential conflict of interest.

## Footnotes

1 https://federalregister.gov/a/2013-01638

2 https://www.fda.gov/drugs/scienceresearch/ucm572617.htm

3 http://www.pharmagtc.org/seapharm

4 www.pharmgkb.org

5 www.cpicpgx.org
